# Strain-level molecular and infection traits are associated with differences in phage efficacy against *Pseudomonas aeruginosa*

**DOI:** 10.1038/s41598-026-64067-w

**Published:** 2026-08-19

**Authors:** Corinna Winkler, Wanqi Huang, Torben Sanders, Xue Peng, Mohammadali Khan Mirzaei, Li Deng

**Affiliations:** 1 Technical University of Munich, TUM School of Life Sciences, Chair of Prevention of Microbial Diseases, Central Institute of Infection Prevention (ZIP), Freising, 85354 Germany; 2 Institute of Virology, Helmholtz Centre Munich - German Research Centre for Environmental Health, Neuherberg, 85764 Munich, Germany; 3 Faculty of Biology, Biocenter, Ludwig Maximilian University of Munich, Planegg-Martinsried, 82152 Munich, Germany; 4 Bavarian Competent Phage Therapy Centre, Munich, Germany; 5 German Center for Lung Research (DZL), Giessen, Germany

**Keywords:** Microbiology, Molecular biology

## Abstract

We identified and characterized a novel lytic phage, HMGUpa1, which exhibited distinct infection efficiency across five characterized *P. aeruginosa* clinical isolates. While all strains were susceptible to the phage, killing efficacy varied significantly, with strain 50071 showing the highest sensitivity. To explore the basis of this variability, we analyzed phage infection dynamics, host genomic features, and transcriptomic responses. Strain-specific differences in gene content and transcriptional profiles, particularly those related to virulence-associated secretion systems, RNA repair, and metabolic reprogramming, were associated with differences in phage efficacy. These findings suggest that differences in phage efficacy arise from a complex interplay of strain-specific molecular, physiological, and infection-related host traits. Together, our results highlight the importance of considering host-specific traits when developing precision-based phage therapy strategies against multidrug-resistant *P. aeruginosa*.

**Supplementary Information:**

The online version contains supplementary material available at 10.1038/s41598-026-64067-w.

## Introduction

The rise of multidrug- and pan-drug-resistant bacteria has made conventional antibiotics ineffective, posing a significant challenge for managing infectious diseases^1^. In addition, the trend has been exacerbated by the slow development of new antibiotics^1,2^. Without effective action, antimicrobial resistance (AMR) could cause up to 10 million deaths annually by 2050^3^. *Pseudomonas aeruginosa* is a leading AMR pathogen and a major cause of hard-to-treat infections worldwide^4,5^. In southern Europe, up to 50% of clinical isolates are classified as multidrug-resistant^6,7^. The World Health Organization (WHO) has identified carbapenem-resistant *P. aeruginosa* as a high-priority pathogen^8^.

This opportunistic bacterium, commonly found in hospital environments, is responsible for approximately 10% of hospital-acquired infections, particularly among immunocompromised patients^4,5^. Its resilience arises from genomic adaptability, a wide range of resistance mechanisms, and its ability to form protective biofilms, which complicates treatment^4,5,9^. Phage therapy, the use of bacteriophages or phages to treat bacterial infections, has re-emerged as a promising alternative to antibiotics, particularly for difficult-to-treat *P. aeruginosa* infections^10–12^.

While phages are promising due to their specificity, low toxicity, and ability to replicate at the infection site, their therapeutic success can vary, even against genetically similar hosts^10–14^. Infection dynamics, including adsorption rate, latency period, and burst size, are known to influence phage efficiency^15,16^. These parameters reflect how efficiently a phage can attach to, infect, and lyse a given host, ultimately shaping its ability to control bacterial populations^17^. However, the molecular basis of this variability remains poorly understood. In this study, we investigated how infection properties, host genomic differences, and host transcriptional responses shape the efficiency of a single lytic phage, HMGUpa1, across five characterized *P. aeruginosa* clinical isolates, aiming to identify key factors that contribute to differences in phage performance across closely related hosts.

## Materials and methods

### Bacterial strains

The bacterial strains used in this study included the clinical *P. aeruginosa* isolates Pa3 (BioSample SAMD00019072), Pa4 (BioSample SAMD00019071), Pa8 (BioSample SAMD00019065), and Pa13 (GenBank accession CP035739.1), obtained from the National Medicines Institute in Warsaw, Poland^18,19^. In addition, *P. aeruginosa* DSM 50071 (GenBank accession CP012001.1) was obtained from the DSMZ collection.

### Growth conditions

Bacterial strains were cultured in lysogeny broth (LB) with shaking at 150 rpm and incubated overnight at 30 °C to reach the stationary phase^20^. Overnight cultures were adjusted to an optical density at 600 nm (OD_600_) of 0.45, corresponding to 2.74 × 10⁹ CFU/ml. These adjusted cultures were then diluted in LB and incubated at 37 °C with shaking for 30 min to stimulate metabolic activity^20^. For plaque assays, LB supplemented with 0.6% agar was used as the soft agar (SA) overlay medium.

### Bacteriophage HMGUpa1

Bacteriophage HMGUpa1 was isolated from sewage water collected at the Oberschleißheim wastewater treatment plant near Munich, Germany, using strain Pa4 as bacterial host. Briefly, the wastewater sample was centrifuged at 4,500 × g for 15 min and subsequently sterile filtered using a 0.22 μm membrane filter (Merck Millipore, Darmstadt, Germany). To amplify the phage, the filtrate was mixed with an equal volume of double-strength LB and an overnight culture of *P. aeruginosa* strain DSM 50,071. This mixture was incubated overnight at 30 °C with continuous shaking at 200 rpm. Following incubation, bacterial cells were removed by centrifugation (4,500 × g, 15 min) and sterile filtration through a 0.22 μm membrane filter. To confirm the presence of phages, the resulting lysate was assessed using a plaque assay^21,22^. Phage lysates were serially diluted 1:10 in LB broth. For each dilution, 100 µL was mixed with 100 µL of the target bacterial strain and 3 mL of 0.6% (w/v) LB soft agar. The mixtures were then poured onto LB agar plates (1.5% w/v agar) and incubated overnight at 30 °C. Individual phages were selected based on plaque morphology. Those that produced clear, non-turbid plaques were classified as virulent. To obtain pure phage isolates, we performed four rounds of plaque purification. Clear plaques were excised and transferred into 800 µL of LB medium. The mixture was vortexed and then incubated at 4 °C for 1 h. After incubation, it was sterile filtered through a 0.22 μm membrane filter. The purified phage, HMGUpa1, was stored in 50% glycerol at − 80 °C or in LB medium at 4 °C^23,24^. Following purification, HMGUpa1 was confirmed to infect all five selected *P. aeruginosa* strains, producing clear plaques on strains 50,071, Pa4, and Pa13, and turbid plaques on strains Pa3 and Pa8 (Table S1).

### Transmission electron microscopy

Phage lysate was propagated and concentrated by centrifugation at 35,000 × g for 3 h. The resulting phage pellet was resuspended in SM buffer (sodium chloride 5.8 g/L, magnesium sulphate heptahydrate 2 g/L, Tris-hydrochloride 6 g/L, pH 7.5), yielding a final titer of 10¹¹ PFU/mL. For imaging, 5 µL of the concentrated phage suspension was applied to a freshly prepared carbon-coated grid. Excess liquid was removed using filter paper, and the grid was negatively stained with 5 µL of 2% uranyl acetate for 30 s. Phage particles were then visualized using a JEM-1400 Plus transmission electron microscope (JEOL, Tokyo, Japan) operated at 80 kV and a magnification of 50,000×.

### Phage killing efficiency

Host bacteria were cultured overnight, adjusted to a concentration of 1 × 10⁸ CFU/mL, and incubated at 37 °C with shaking for 30 min to stimulate metabolic activity. HMGUpa1 was then added at multiplicities of infection (MOIs) of approximately 1, 10, 50, or 100. Infection kinetics were monitored by measuring optical density at 600 nm (OD₆₀₀) using a BioTek Epoch 2 Microplate Spectrophotometer (Agilent Technologies, Santa Clara, USA). Readings were taken immediately after phage addition and subsequently at 30-minute intervals over a 24-hour period. During this time, the plates were incubated at 37 °C with continuous shaking at 200 rpm. Growth curves were generated by plotting baseline-adjusted OD₆₀₀ values against time. To quantify overall growth inhibition resulting from phage treatment, the area under the curve (AUC) for each condition was calculated using GraphPad Prism (v10.5.0). Lower AUC values reflected greater phage efficacy. Killing efficiency (KE) was calculated using the following formula: KE (100%) = [(OD_positive control_ - OD_treatment_) / OD_positive control_] × 100. Statistical analysis of AUC values was performed using one-way analysis of variance (ANOVA) followed by Tukey’s multiple-comparison test to assess the effect of different multiplicities of infection (MOIs) within each bacterial strain. All assays were conducted in 96-well microtiter plates and performed in three biological replicates.

### Phage infection property

#### Adsorption assay

The adsorption rate of phage HMGUpa1 was measured independently following the method described by Hyman and Abedon^25^. *P. aeruginosa* strains (50071, Pa3, Pa4, Pa8, and Pa13) were cultured overnight, adjusted to 2.74 × 10⁸ CFU/mL, and incubated at 37 °C with shaking for 30 min to stimulate metabolic activity. Phages were added at a multiplicity of infection (MOI) of 0.001 and incubated at 37 °C with gentle shaking throughout the experiment. Samples were collected every minute for a total of 8 min, sterile filtered to remove bacteria, and stored on ice. Phage titers (PFU/mL) were determined by plaque assay. The adsorption rate constant (*k*) was calculated using the equation: *k* = − ln (P/P_0_) / Nt, where *P* and *P₀* are the final and initial phage concentrations, *N* is the bacterial density, and *t* is the incubation time in minutes^25^. Three biological replicates were conducted for each host strain.

#### One-step growth curve

The latency period (LP) and burst size (BS) of phage HMGUpa1 were determined using a standard one-step growth curve protocol, as outlined by Hyman and Abedon^25^. First, *P. aeruginosa* host strains (50071, Pa3, Pa4, Pa8, and Pa13) were cultured overnight and adjusted to a concentration of 2.74 × 10⁸ CFU/mL. After incubating at 37 °C for 30 min with shaking to stimulate metabolic activity, phages were introduced at an MOI of 0.01. The mixtures were then incubated at 37 °C with continuous shaking, and samples were collected every 10 min. For strain 50071, sampling continued for 90 min to capture the complete infection cycle, which revealed a latency period of 10 min. Sampling of the remaining host strains was limited to 30 min, which was considered sufficient for the comparative assessment of infection properties across the different host strains. Phage titers at each time point were measured using a plaque assay (PFU/mL). The latency period was defined as the time from phage addition to the first increase in titer, indicating the beginning of lysis. Burst size was calculated by dividing the maximum phage titer after burst by the number of phages adsorbed. All experiments were conducted in three biological replicates for each host strain.

### Phage genome annotation and characterization

The genome of phage HMGUpa1 was annotated using Pharokka (v1.7.2). Genome characterization was performed using Sphae^26^, which was used to screen for lysogeny-associated genes, antimicrobial resistance genes, virulence factor genes, defense genes, and CRISPR spacers. The predicted replication cycle of HMGUpa1 was assessed using Bacphlip^27^.

### Comparative genomics

Comparative genomic analyses were conducted to assess the relationship between phage HMGUpa1 and its bacterial hosts. Average amino acid identity (AAI) and average nucleotide identity (ANI) values were calculated using the AAI and ANI calculators (http://enve-omics.ce.gatech.edu/aai/ and http://enve-omics.ce.gatech.edu/ani/). Protein sequences encoded by the phage and bacterial genomes were extracted, and a non-redundant gene catalog was generated using MMseqs2 with a 70% sequence identity cutoff^28^. Protein-sharing profiles among the phage and host genomes were then derived from the clustering results and visualized using principal component analysis (PCA) and UpSet plots^29^. Prophage regions in bacteria genomes were predicted using PHASTER^30^ and aligned with the phage genome to assess similarity using BLAST^31^ (v2.13.0). Functional annotation of genes was performed using the KEGG database^32^(https://www.genome.jp/kegg/pathway.html) and kofamkoala (https://www.genome.jp/tools/kofamkoala/*).* Bacterial genomes were visualized using BRIG^33^ (v0.95) (https://brig.sourceforge.net/) and Clinker^34^ (v0.0.23). The phage HMGUpa1 has been sequenced and deposited in NCBI under accession number OQ338182.

### RNA extraction from phage-infected bacteria

Overnight cultures of *P. aeruginosa* strains (50071, Pa3, Pa4, Pa8, and Pa13) were diluted in LB medium and incubated at 37 °C until reaching an optical density (OD₆₀₀) of 0.3. All cultures were standardized to 1.37 × 10⁸ CFU/mL. Each strain was infected with phage HMGUpa1 at an MOI of 50. Samples of 10 mL were collected at 0, 5, 10, and 20 min post-infection, centrifuged at 7,000 × g for 30 min, and processed for total RNA extraction using the QIAGEN RNeasy PowerMicrobiome Kit. T0, collected immediately after phage addition, was used as the reference condition for differential expression analysis to provide a common baseline at the onset of infection and to account for any RNA introduced with the phage preparation. The selected intervals were designed to capture early transcriptional responses to phage infection, including adsorption, and early gene expression. All experiments included three biological replicates. Library preparation and sequencing were carried out at the Dresden-concept Genome Center following rRNA depletion, using the Illumina NovaSeq 6000 platform with a NovaSeq S4 v1.5 4XP flow cell (200 cycles).

### RNA-seq analysis

Bacterial genomes were annotated using Bakta (v1.9.4) with full database PHANOTATE (v1.5.1). Gene annotation GTF files were obtained from GeneBank files of each genome. Raw Illumina reads (FASTQ format) were first assessed for quality using FastQC (v0.12.1), evaluating metrics such as per-base sequence quality, overall sequence quality, GC content, and adapter content. A combined quality control report was generated using MultiQC (v1.17). Adapter trimming and quality filtering (removal of reads < 25 bp) were performed using Fastp (v0.23.4), producing cleaned FASTQ files along with JSON and HTML quality reports. Cleaned reads were mapped to their respective reference genomes using Bowtie2 (v2.5.3), with reference indices built beforehand. The resulting SAM alignment files were converted to sorted BAM format using SAMtools (v1.20). Gene-level quantification was then performed using FeatureCounts, which used the BAM files and GTF annotation files to generate count matrices. The processed data were used for differential expression analysis in the statistical language R using the DESeq2 package (PyDESeq2, v1.44.0) and Wald test for standard differential analysis. A DESeq2 dataset was created and the DESeq2 analysis was run. When comparing all samples to each other, such as all bacterial genomes or phage genome across samples, the all_vs_all parameter was used, whereas for comparison within the same genome, one_vs_all was used,  with T0 serving as the control and T5, T10, and T20 as the comparison time points. Differentially expressed genes identified by DESeq2 were filtered using the following criteria: adjusted p-value (padj) < 0.05 and absolute log2 fold change ≥ 3. Negative log2FC values corresponded to higher expression at T5, T10, and T20 relative to T0, whereas positive log2FC values indicated lower expression relative to T0. Genes with missing values were filtered out. If no genes meeting the filtering criteria could be identified, the analysis was stopped. The remaining genes were sorted by p-value. Next, the top 50 differentially expressed genes were selected, aggregated and normalized, creating a subset matrix, aggregating replicate counts for each condition and using Z-score to standardize for comparison across samples. The heatmap generation was performed in the package Pheatmap (v1.0.12) and RColorBrewer (v1.1.3). PCA plots and graphs were created using the tools Seaborn (v0.13.2) and Plotly Express.

### Functional prediction of genes with unknown function

Functional prediction of hypothetical, meaning currently unannotated, phage proteins was completed with DeepFRI’s online tool using a threshold of 0.5 to ensure a significant prediction (https://beta.deepfri.flatironinstitute.org/AboutUs*).* Protein-protein interaction (PPI) predictions were subsequently run with SpeedPPI’s some versus some mode (https://github.com/patrickbryant1/SpeedPPI*).* The selection criterion for bacterial proteins included in this analysis was a log2FC value of ± 3. To yield the most meaningful and trustworthy predictions, a threshold of 0.5 for the predicted DockQ (pDOCKQ) score and the average interface predicted local distance difference test (pLDDT) was applied. pDOCKQ is a predicted quality measure for protein docking models (https://www.nature.com/articles/s41467-022-28865-w*).*

## Results

**Fig. 1:**
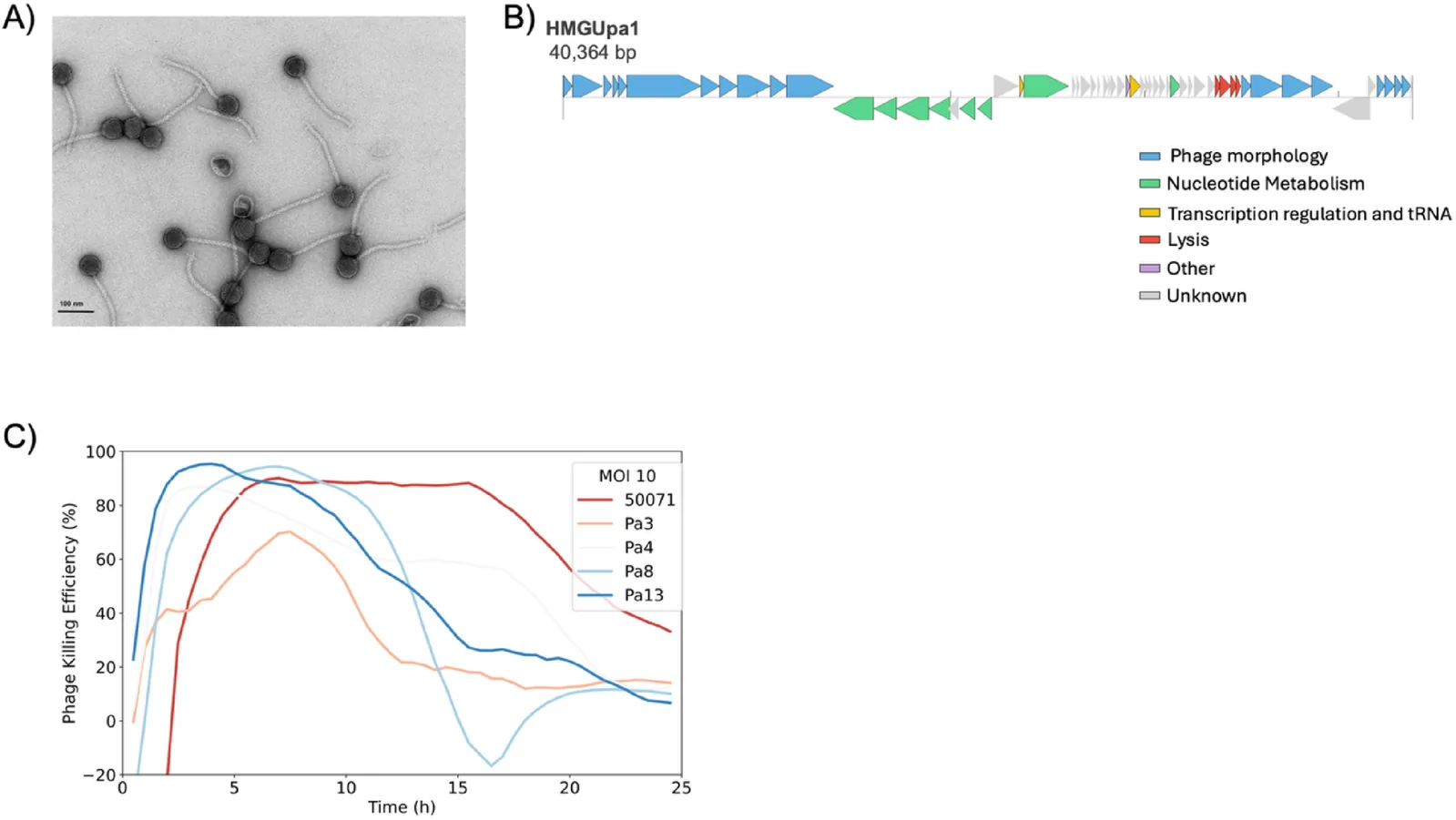
TEM image of phage HMGUpa1 (**A**); genome map of HMGUpa1 showing 57 CDSs (**B**); phage killing efficiency against five *P. aeruginosa* strains (**C**).

To investigate how phage infection dynamics vary across different *P. aeruginosa* hosts, we selected phage HMGUpa1 based on its ability to efficiently infect five genetically distinct strains. Transmission electron microscopy (TEM) revealed an icosahedral capsid approximately 60 nm in diameter and a long, non-contractile tail (~ 200 nm), characteristic of a Siphovirus morphotype within the class *Caudoviricetes* (Fig. 1A)^35,36^. De novo genome assembly yielded a single linear double-stranded DNA (dsDNA) contig of 40,364 bp, with a G + C content of 54.60% and 57 predicted coding DNA sequences (CDSs) (Fig. 1B). Of these, 33 CDSs were functionally annotated and grouped into modules related to phage structure, nucleotide metabolism, transcriptional regulation, tRNA biology, and host lysis; the remaining CDSs encoded hypothetical or uncharacterized proteins. In addition, Bacphlip predicted HMGUpa1 to be a virulent phage, and Sphae analysis did not identify lysogeny-associated genes, antimicrobial resistance genes, or virulence factors, supporting this prediction.

To assess the killing efficacy of HMGUpa1 across these hosts, we tracked bacterial growth following infection at MOIs of 1, 10, 50, and 100 (Fig. S1; Table S2). In all strains, AUC values of infected cultures were consistently lower than untreated controls, indicating successful infection. The strongest bacterial growth suppression was observed in strain 50071, with AUC reductions of approximately 70% across all MOIs. Although ANOVA analysis did not reveal statistically significant differences between MOIs (F = 1.57, *P* = 0.24), higher MOIs showed a trend toward increased efficacy compared to MOI 1. MOI 10 was selected for subsequent experiments, as it achieved comparable suppression without requiring excessive phage input. The killing efficiency of HMGUpa1 varied considerably between host strains (Fig. 1C). Strain 50071 exhibited the highest and most sustained reduction in bacterial growth, reaching ~ 90% killing efficiency by 15 h post-infection. Strains Pa4, Pa13, and Pa3 exhibited lower killing efficiencies of approximately 60%, 30%, and 20%, respectively, with Pa3 and Pa13 showing initial suppression followed by partial recovery, indicative of bacterial regrowth. In contrast, Pa8 showed near-complete resistance, with killing efficiency remaining close to 0% and eventually declining below baseline (< 0%), consistent with pronounced bacterial regrowth. These findings underscore the host-specific nature of phage susceptibility, with strain 50071 emerging as the most permissive host for HMGUpa1 infection.

Given the significant differences in HMGUpa1 killing efficiency across *P. aeruginosa* strains, we investigated whether host-specific variation in infection properties could account for this heterogeneity. Phage efficacy is influenced not only by host range but also by infection parameters such as adsorption rate, latency period, and burst size, which together shape the pharmacodynamics of phage activity. To this end, we quantified these infection parameters for HMGUpa1 across all five hosts and observed significant differences. The latency period was approximately 10 min for strains 50071, Pa3, Pa8, and Pa13, whereas it extended to over 30 min for strain Pa4 (Fig. S3, Table S3). While the burst sizes were generally small (1.07–1.54 per infecting phage) among hosts, the adsorption rate constants (*k*) ranged from 9.29 × 10⁻¹¹ to 3.46 × 10⁻¹⁰, with 50071 exhibiting the slowest adsorption rate and Pa3 the fastest (Figs. S2 and S3, Tables S3 and S4). To evaluate how these properties relate to phage efficacy, we calculated Kendall rank correlation coefficients between each parameter and the AUC values of the killing efficiency (AUC). We found a positive correlation between AUC and latency period (τ = 0.63) and a negative correlation with adsorption rate (τ = -0.8).

**Fig. 2:**
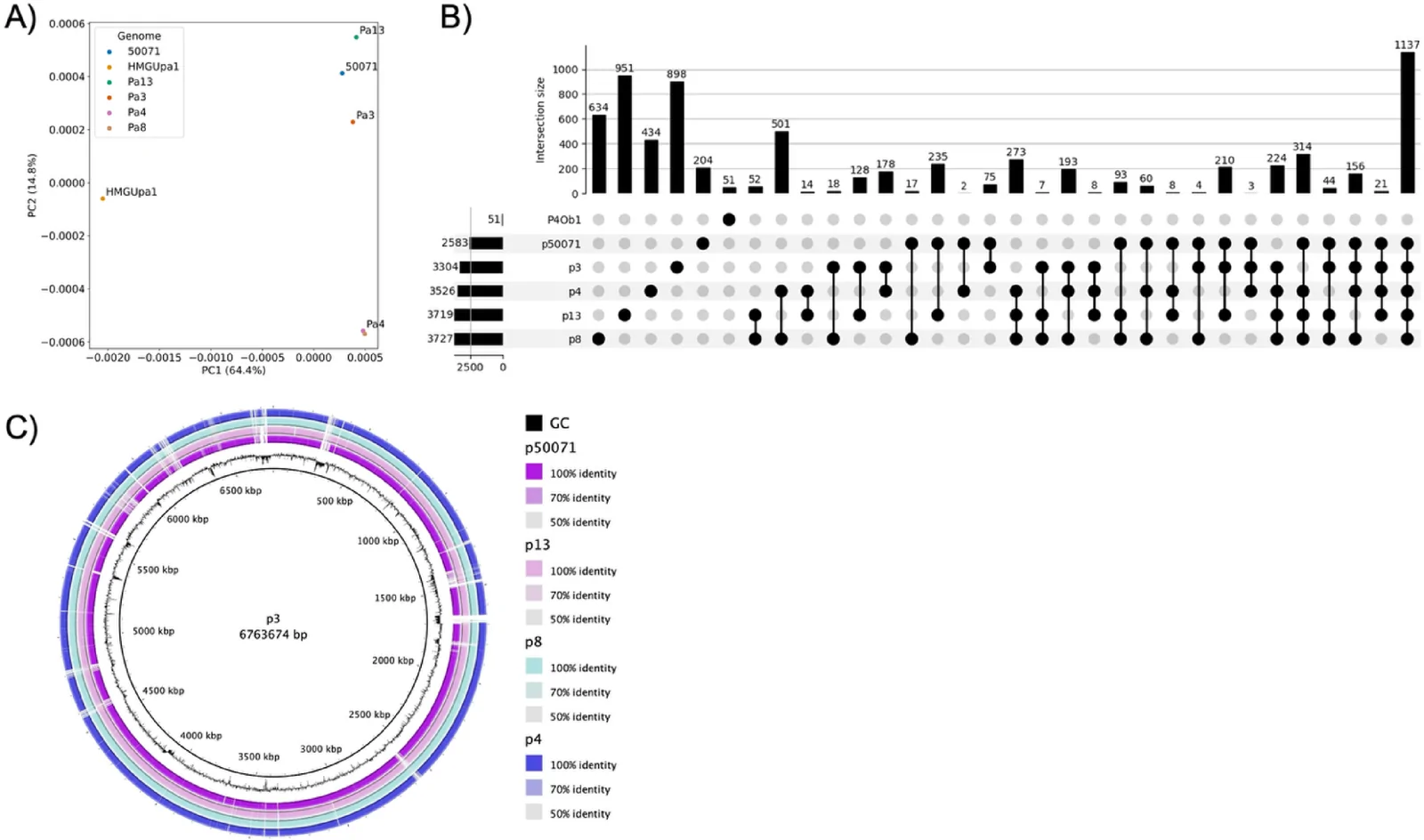
Principal component analysis (PCA) of gene expression following infection (**A**); UpSet plot showing shared and unique protein clusters among phage and hosts (**B**); circular representations of the five host bacterial genomes (**C**).

While latency period may influence phage efficacy, the absence of consistent trends across all infection parameters suggests additional host-specific factors contribute to susceptibility. To explore this, we examined genomic similarities among the *P. aeruginosa* strains. ANI and AAI analyses revealed that all strains shared high genomic similarity (ANI > 99%, AAI > 98%), with Pa4 and Pa8 being the most similar to each other and most distinct from the remaining strains (Fig. 2 and Table S5). HMGUpa1 shared no detectable ANI with its hosts, and the highest AAI observed was only 35.05% with strain 50071. We next assessed prophage content, which ranged from five to ten predicted regions per genome. Pa8 harbored the highest number (10), followed by Pa3 (9), Pa13 (8), 50071 and Pa4 (5 each). Comparative genomics (Fig. S4) revealed three prophage regions with sequence similarity to HMGUpa1. Further analysis in Pa3 identified two BLAST hits within a prophage region (4725–56361 bp), one corresponding to a “major capsid protein 13-like” gene and another outside of a predicted coding sequence.

To assess host-specific functional differences, we used KEGG annotations to compare gene content of individual strains, which revealed distinct functional profiles among them (Fig. 2 and Table S6). Strain 50071 carried the fewest unique genes, primarily linked to metabolism, while other strains harbored unique genes across a broader range of pathways, including environmental and genetic information processing. HMGUpa1 encoded 51 unique genes, with no overlap with any host. Pa3 and 50071 had the highest number of unannotated unique genes, with 635 and 154, respectively, possibly hindering the identification of additional functional differences. KEGG annotations assigned unique genes to six major functional categories (Table S6), with metabolism-related genes being the most prevalent. In strain Pa13, 96 unique genes were associated with metabolism, including 24 for amino acid metabolism and 18 for cofactor and vitamin metabolism.

**Fig. 3:**
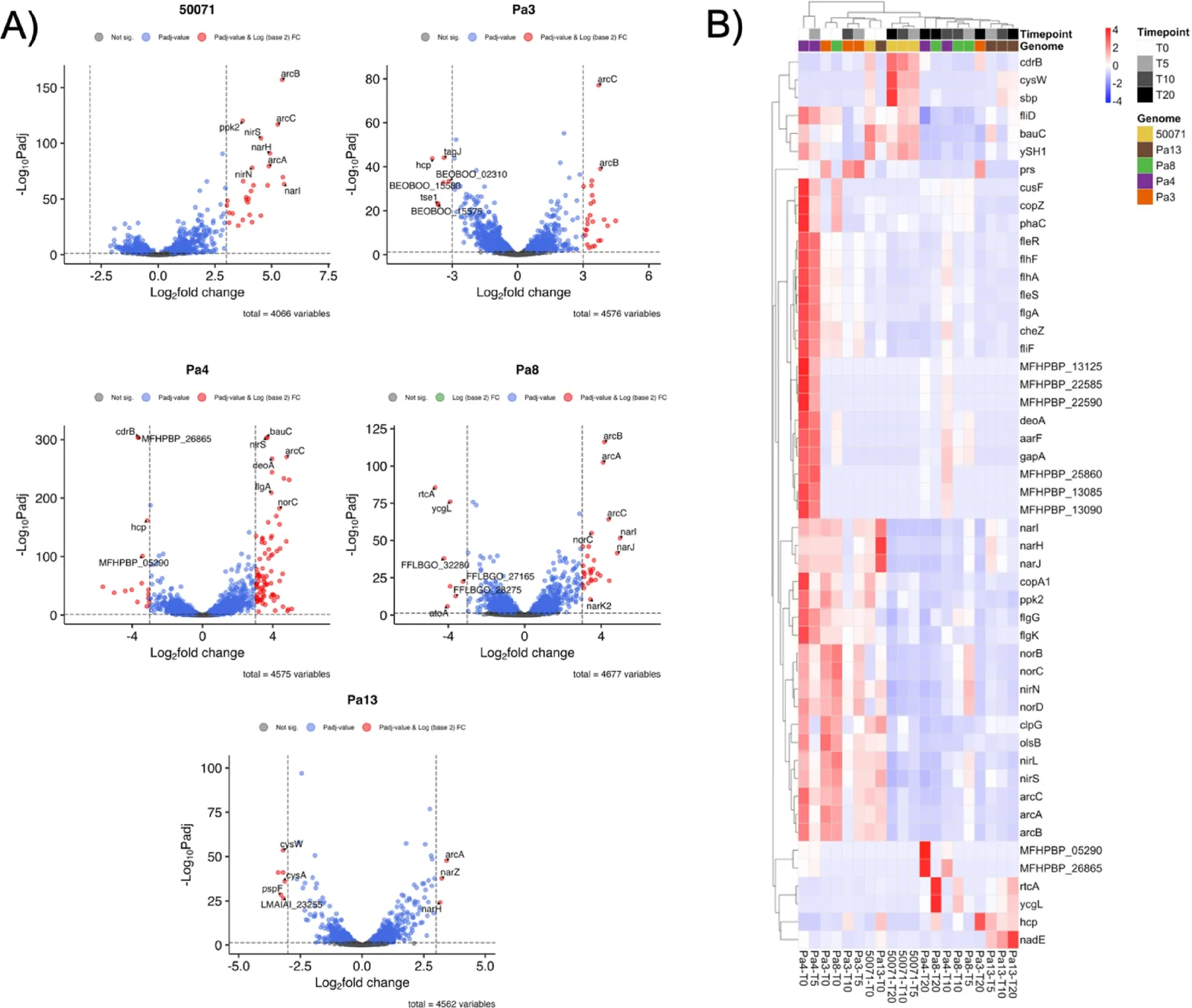
(**A**) Volcano plots showing differentially expressed genes (log2FC ≥ 3, *p-adj* < 0.05) across five *P. aeruginosa* strains 20 min post-infection (MOI = 50). (**B**) Heatmap of Z-score normalized gene expression, revealing distinct clustering between responsive and suppressed host profiles.

Building on these genomic differences, we conducted time-resolved transcriptomic profiling at 0, 5, 10, and 20 min post-infection (MOI = 50) to capture host regulatory responses across early to late infection stages. Analysis of the top 20 differentially expressed genes in each strain revealed a common feature across the five strains: downregulation of genes involved in nitrate respiration and arginine metabolism, including *narI*,* narJ*,* narH*,* narZ*,* arcA*,* arcB*,* and arcC*, while infection-induced gene expression was highly strain-specific. The Volcano plots showed that strain 50071, the most susceptible host, experienced strong early downregulation of genes involved in arginine metabolism, polyphosphate metabolism, and anaerobic respiration (e.g., *narI*,* arcB*,* arcA*,* arcC*,* narH*,* nirS*,* and nirM*) (Fig. 3A; Tables S7, S8). Notably, no comparable set of significantly upregulated genes was detected in 50071. In contrast, the less susceptible strains Pa3, Pa4, Pa8, and Pa13 exhibited more gene upregulations, particularly at later infection stages. At 20 min post-infection, Pa3 exhibited late induction of secretion-associated genes, such as *tse1* and *hcp*, potentially linked to T6SS-related functions, suggesting a robust physiological response to phage infection. Pa4 upregulated the gene, *cdrB*, related to secretion and biofilm-associated functions. Pa8 showed upregulation of *rtcA*,* rtcB*, and *atoA*, suggesting that late infection was associated with RNA repair-related processes and metabolic adjustments. In Pa13, *cysT*,* cysW* and *cysA* were upregulated, indicating increased expression of sulfate transport-related genes, while upregulated *pspF* suggested a possible envelope stress-related response (Fig. 3A, Table S7). These results suggest that successful infection by HMGUpa1 coincides with a transcriptionally suppressed state that lacks immune activation, while resistant strains engage broader physiological adjustments to manage infection stress. The hierarchical clustering (Fig. 3B) revealed strain-specific transcriptional responses to phage infection. While T0 samples clustered closely across strains, strain 50071 diverged from the remaining hosts following infection, whereas Pa3, Pa4, Pa8, and Pa13 exhibited more similar time-dependent transcriptional responses.

**Fig. 4:**
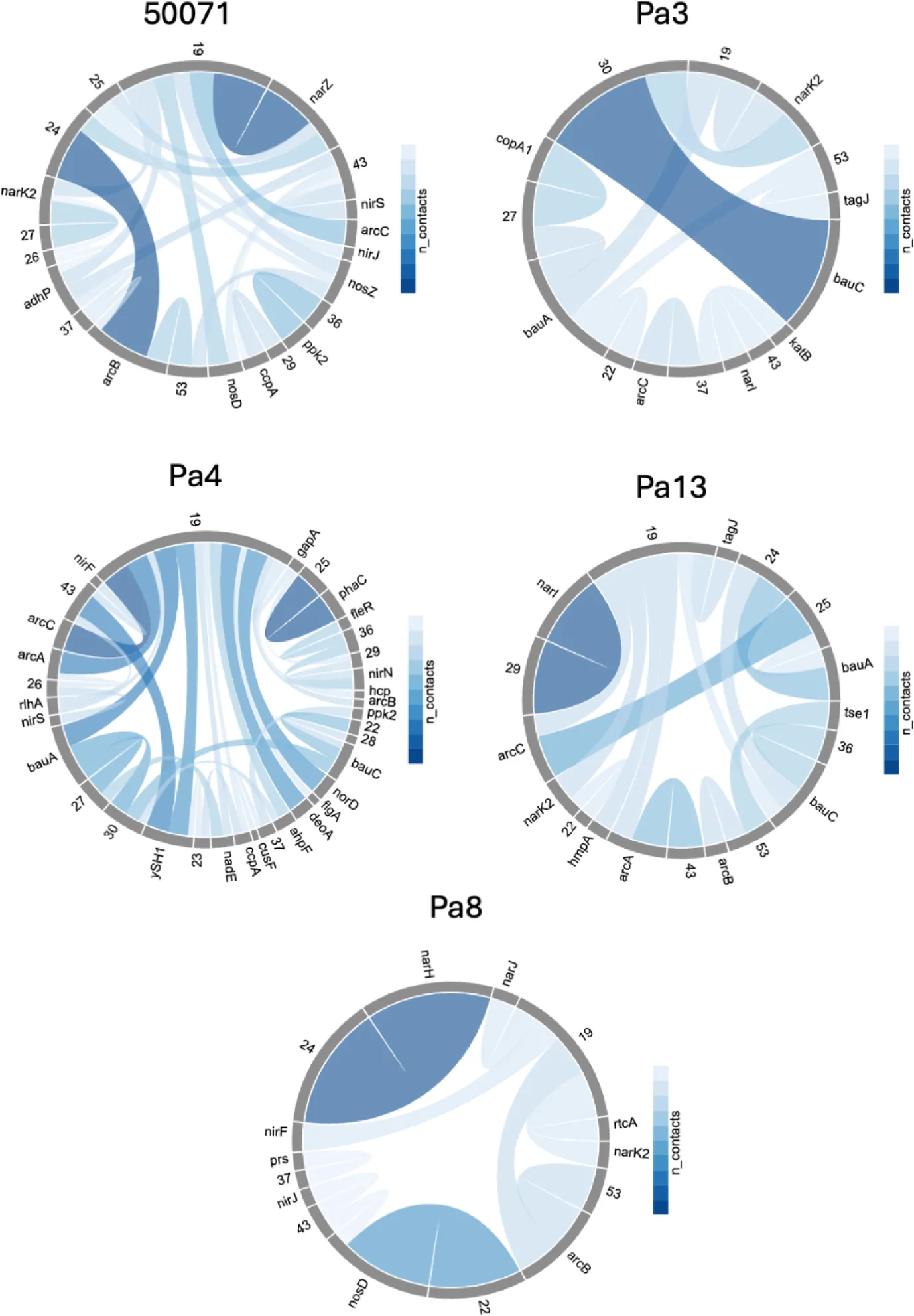
Chord plots of predicted high-confidence phage–host protein interactions per strain.

To further explore the molecular basis of host-specific phage efficacy, we examined predicted protein-protein interactions (PPIs) between phage HMGUpa1 and host proteins in strains 50071 and Pa8—representing the highest and lowest killing efficiencies, respectively (Fig. 4). High-confidence PPIs were filtered for bacterial proteins showing log₂ fold changes ≥ ± 3.0 (Tables S9, S10). Strain 50071 exhibited 24 filtered interactions, primarily involving proteins in nitrogen and arginine metabolism (*nosD*,* narK2*, *nirS*,* narZ*,* arcC*), as well as carbon (*adhP*), phosphate (*ppk2*), and oxidative stress pathways (*ccpA*), suggesting a broad phage engagement with core metabolic systems. In contrast, Pa8 showed only 11 interactions, mostly targeting proteins involved in nitrogen metabolism, nucleotide biosynthesis and RNA repair (*rtcA*,* prs*), along with other components of replication and transcription. These results suggest that in 50071, phage–host interactions are more metabolically oriented, particularly toward anaerobic energy production, whereas in Pa8, they center on bacterial replication and transcription processes, possibly reflecting a stress-induced host response.

## Discussion

*P. aeruginosa* is a metabolically adaptable and clinically significant pathogen that poses a major therapeutic challenge due to its multidrug resistance^4–9^. While phage therapy is being explored as a possible alternative to traditional antibiotics, variations in phage effectiveness, even among closely related strains, may have been underestimated. This issue is specifically relevant for pre-formulated phage cocktails, which often include selected phages that have not been thoroughly tested against the specific strains infecting individual patients^37–39^.

In this study, we characterized the infection dynamics of the lytic phage HMGUpa1 across five *P. aeruginosa* strains. Despite the high genomic similarity among these strains, HMGUpa1 displayed significantly variable killing efficiencies, ranging from approximately 90% in strain 50071 to nearly zero in strain Pa8. These differences cannot be fully explained by infection parameters such as latency period, burst size, or other standard metrics. While we observed some correlations, for instance, a shorter latency period was linked to increased killing efficiency, these trends were not consistent across all strains and conditions.

To explore other potentially contributing factors, we examined the genomic profiles of the host strains. KEGG pathway analysis revealed that strain 50071 harbored fewer unique genes than the others, with most annotated in core metabolic pathways. In contrast, the remaining strains exhibited broader functional diversity across pathways associated with cellular processes, environmental and genetic information processing, human diseases, and organismal systems. However, no specific genetic elements could be directly associated with the observed differences in phage susceptibility. Nevertheless, these genomic differences may still be relevant to phage–host interactions, as suggested by previous studies^40,41^. In addition, the comparative genomic analysis showed a similarity between HMGUpa1 and strain Pa3, which exhibited a killing efficiency of only 20%. The observed genomic similarity raises the possibility of superinfection exclusion (SIE), a hypothesis that requires experimental validation. SIE is a mechanism whereby phage-infected cells resist subsequent infection by related phages, thereby preventing productive replication^16,42^. In *P. aeruginosa*, SIE has been linked to deficiency of type IV pilus (T4P) function, which is often essential for phage attachment and entry^42,43^.

To gain deeper insight into the molecular mechanisms underlying the observed differences in phage infection efficiency, we conducted time-resolved transcriptomic profiling of all five *P. aeruginosa* strains during HMGUpa1 infection. The different gene expressions suggest that phage infection triggered both shared and strain-specific transcriptional changes. The repeated downregulation of nitrate respiration and arginine metabolism associated genes across the strains indicates a common host response with repression of respiratory and energy metabolism-related functions following phage exposure, which may potentially facilitate effective phage replication by redirecting host resources and reducing basal activity of energy-demanding defense systems. This observation is in line with previous findings demonstrating that decreasing host metabolic and defense functions can enhance phage infection^44–46^.

Notably, the most susceptible strain, 50071, exhibited a predominantly downregulation-driven response with limited transcriptional induction, whereas the less susceptible strains displayed highly strain-specific transcriptional activation. These differences suggest that successful HMGUpa1 infection is associated with a relatively suppressed host transcriptional response, whereas reduced phage efficacy is associated with activation of diverse physiological pathways. For example, induction of T6SS-associated genes in Pa3 and biofilm-associated secretion genes in Pa4 may reflect adaptive responses to infection-associated stress, consistent with previous observations linking these pathways to environmental stress and phage exposure^47,48^. Likewise, activation of RNA repair-associated genes in Pa8 and sulfate transport- and envelope stress-related genes in Pa13 suggests that different hosts engage distinct physiological programs in response to phage infection. Together, these observations suggest that transcriptional plasticity, rather than a single conserved defense mechanism, is associated with differences in phage infection outcomes.

Taken together, these findings indicate that phage efficacy is associated not only with infection kinetics but also with a complex interplay of strain-specific genomic, regulatory, and physiological traits. Bacterial resistance can act at any stage of the phage replication cycle, from receptor recognition to virion release^49,50^. This is consistent with our observation that phage genes involved in infectivity, replication, and lysis were more strongly expressed in strain 50071 than in Pa8, suggesting that Pa8 may potentially restrict phage replication. Supporting this, PPI analyses showed that Pa8 was enriched in host replication and transcription processes, potential bottlenecks for phage propagation. In contrast, PPIs at 50071 were associated with anaerobic energy production, which may indicate increased support for phage gene expression. However, these predicted interactions require experimental validation before mechanistic conclusions can be drawn. In addition, the absence of time-matched, uninfected controls in our transcriptomic analysis limits the formal attribution of all transcriptional changes exclusively to phage infection, even though all strains were analyzed under identical experimental conditions throughout the early stages of infection.

Our findings reveal that phage efficacy against *P. aeruginosa* is shaped by a complex interplay of strain-specific molecular, transcriptional, and infection-related factors. Even closely related strains showed distinct responses to the same phage, highlighting the complexity of phage–host interactions and the limitations of generalized therapeutic approaches. Incorporating molecular profiling into clinical practice could help match phages to specific bacterial targets, improving therapeutic success.

## Supplementary Information

Below is the link to the electronic supplementary material.

Supplementary Material 1

Supplementary Material 2

## Data Availability

The sequencing data generated in this study have been submitted to public repositories. The phage genome is available under accession number (OQ338182), and the transcriptome data are available under accession number (PRJNA1277130). All code used for data analysis is available at: [https://github.com/tsanders00/winkler_2025_phage_efficacy_p_aeruginosa].
