# Supplementary material for: Strain-level molecular and infection traits are associated with differences in phage efficacy against Pseudomonas aeruginosa
Source: Sci Rep. 2026 Aug 19;16:26054. doi: 10.1038/s41598-026-64067-w (PMC13490528; doi:10.1038/s41598-026-64067-w)
Supplement: Supplementary file 1 [file 41598_2026_64067_MOESM1_ESM.docx]

## **Supplementary Figures**

**Figure S1.** Growth dynamics of five different *P. aeruginosa* strains (50071, Pa3, Pa4, Pa8, and Pa13) after phage infection by HMGUpa1 at MOI 1, 10, 50, and 100 and the respective bacterial control as reference.

**Figure S2.** Adsorption kinetics of HMGUpa1. The concentration of free phages after infection of 50071, Pa3, Pa4, Pa8, and Pa13 was quantified using plaque assay. Bacteria overnight cultures were infected with phage HMGUpa1 at MOI of 0.001 and incubated at 37°C for 8 min. The data are presented as the mean ± SD of three independent replicates.

**Figure S3.** One-step growth curve of HMGUpa1 infecting five different *P. aeruginosa* host strains 50071, Pa3, Pa4, Pa8, and Pa13. Bacterial overnight cultures were inoculated with HMGUpa1 at MOI of 0.01 and incubated at 37°C for 30 minutes or 90 minutes. The phage concentration was assessed after every 10 minutes using plaque assay. The latent period (LP), rising period (RP), and burst size (BS) for HMGUpa1 in each strain are indicated by the grey dotted lines. The data are presented as the mean ± SD of three independent replicates.

**Figure S4.** Clinker visualization revealed three prophage regions with sequence similarity to HMGUpa1.

**Figure S5.** Differentially expressed phage genes measured immediately, 5, 10 or 20 minutes after infection of five different *P. aeruginosa* hosts (50071, Pa3, Pa4, Pa8, and Pa13).
